# Supplementary material for: Low excitatory innervation balances high intrinsic excitability of immature dentate neurons
Source: Nat Commun. 2016 Apr 20;7:11313. doi: 10.1038/ncomms11313 (PMC4843000; doi:10.1038/ncomms11313)
Supplement: Supplementary Information — Supplementary Figures 1-6, Supplementary Table 1 and Supplementary References. [file ncomms11313-s1.pdf]

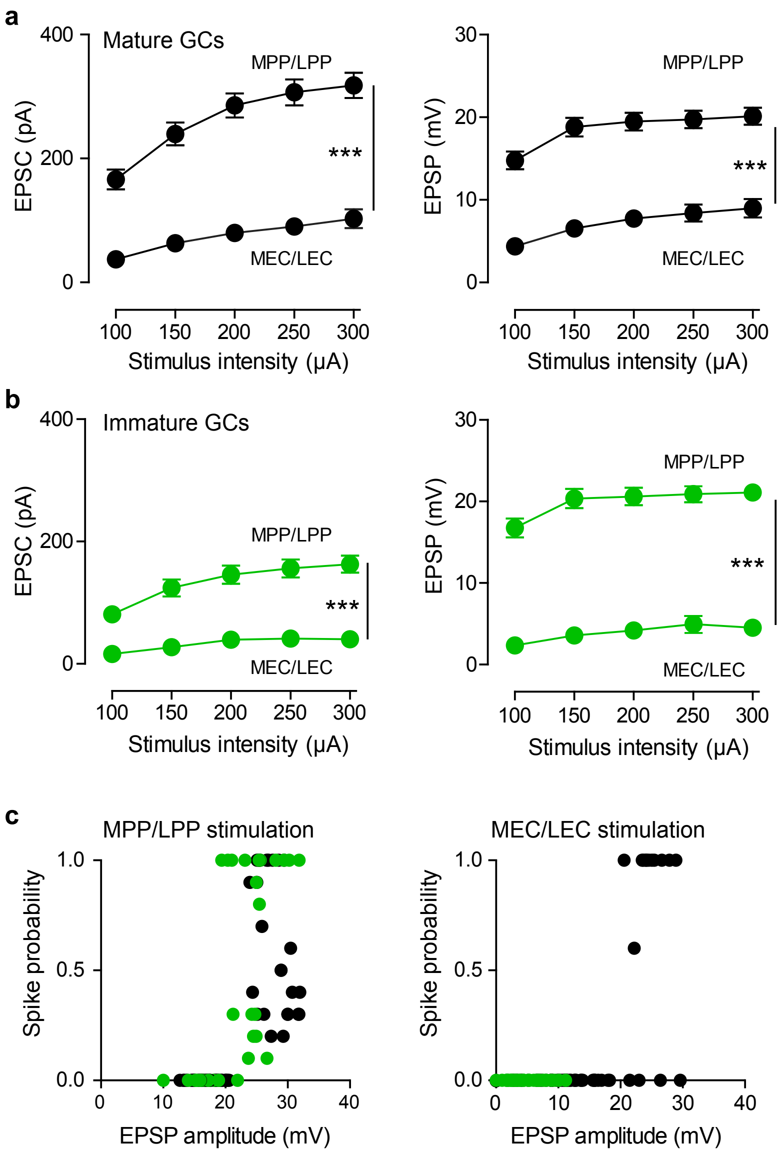

Supplementary Figure 1. EC stimulation is a "weak" stimulus paradigm compared to molecular layer stimulation.

a. Left, in mature GCs, MEC/LEC stimulation generated significantly smaller EPSCs than MPP/LPP stimulation. Repeated-measures two-way ANOVA, n = 18-31 cells, factor pathway, $F_{(1,47)} = 57$, $p < 0.0001$; factor stimulus, $F_{(4,188)} = 76$, $p < 0.0001$;

interaction, $F_{(4,188)} = 14$, $p = 0.0001$. Right, MEC/LEC stimulation also generated significantly smaller EPSPs than MPP/LPP stimulation. Repeated-measures two-way ANOVA, n = 18-31, factor pathway, $F_{(1,47)} = 57$, $p < 0.0001$; factor stimulus, $F_{(4,188)} = 63$, $p < 0.0001$.

b. Left, in immature GCs, MEC/LEC stimulation evoked significantly smaller EPSCs than MPP/LPP stimulation. Repeated-measures two-way ANOVA, n = 18-31 cells, factor pathway, $F_{(1,47)} = 4$, $p < 0.0005$; factor stimulus, $F_{(4,188)} = 63$, $p < 0.0001$; interaction, $F_{(4,188)} = 21$, $p < 0.0001$. Right, EPSPs in immature GCs evoked by MEC/LEC stimulation were also smaller compared to the MPP/LPP stimulation. Repeated-measures two-way ANOVA, n = 18-31 cells, factor pathway, $F_{(1,47)} = 132$, $p < 0.0001$; factor stimulus, $F_{(4,188)} = 25$, $p < 0.0001$; interaction, $F_{(4,188)} = 4$, $p < 0.0001$. Only subthreshold EPSPs in mature GCs were included in this analysis and MPP/LPP data is from[1]. Symbols represent mean ± SEM.

c. Comparison of EPSP amplitude and spiking probability reveals that MEC/LEC stimulation generates EPSPs in immature GCs (green) that are too small to achieve spike threshold. In contrast, EPSPs in immature GCs evoked by molecular layer stimulation (MPP/LPP) are sufficient to achieve threshold. EPSPs in mature GCs (black) are sufficient to generate spikes in both stimulation paradigms. EPSP amplitudes were measured from individual subthreshold EPSPs with the average spiking probability calculated from 10-20 trials at the same stimulating intensity. Data from MPP/LPP stimulation is from[1], n = 32 cells; data from MEC/LEC stimulation is from Figure 2, n = 32 cells.

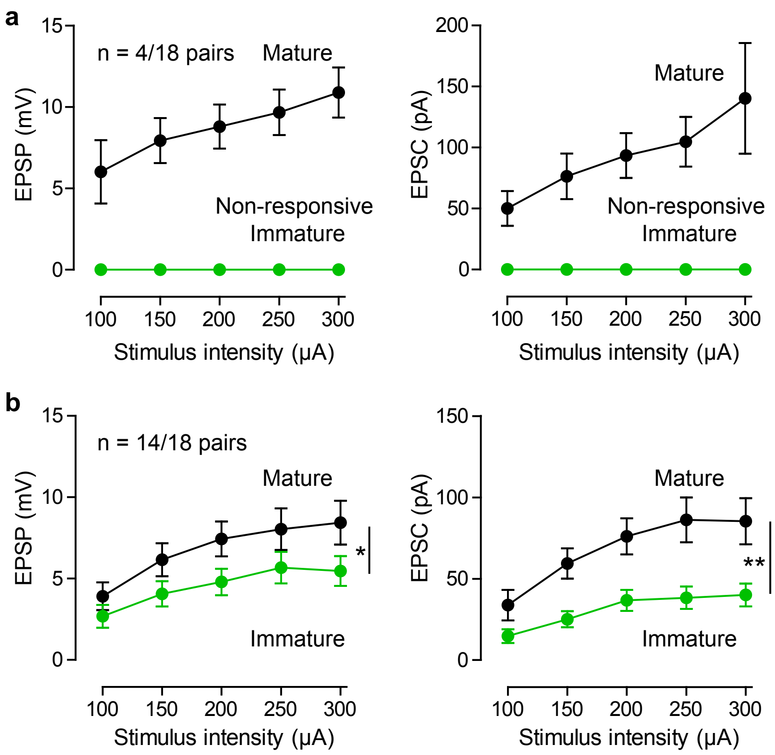

Supplementary Figure 2. Reduced excitatory drive is apparent when non-responsive immature GCs are excluded from analysis.

a. In the data set of sequential mature and immature GC recordings (Figure 2d,e), 22% of immature granule cells (4/18) did not have measurable EPSPs or EPSCs.

b. The difference between mature and immature EPSPs and EPSCs remained significant when the non-responding pairs shown in (A) were excluded from analysis. Repeated-measures two-way ANOVA, n = 14 pairs: EPSPs: factor cell age, $F_{(1,26)} = 4$, *$p = 0.045$; factor stimulus, $F_{(4,104)} = 26$, $p < 0.0001$; EPSCs: factor cell age, $F_{(1,26)} = 11$, **$p = 0.002$; factor stimulus, $F_{(4,104)} = 19$, $p < 0.0001$.

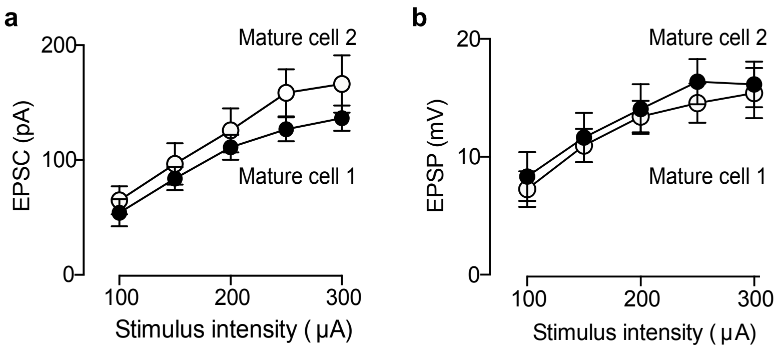

Supplementary Figure 3. Sequential recordings from mature GCs show no difference in excitatory drive.

There was no difference in EPSCs (left) and EPSPs (right) during sequential recordings from two mature GCs. The location of the stimulating electrodes were optimized for mature GC #1 (solid symbols), with subsequent recording from neighboring mature GC #2 (open symbols) in response to the same MEC/LEC stimulation (n = 9 pairs of mature GCs). Responses increased with stimulation intensity, but were not different between the first and second recorded mature GC. These results confirm that the order of cell recording does not influence the amplitude of EPSCs and that mature GCs sample equally from the same pool of stimulated axons . Repeated-measures two-way ANOVA, EPSC: factor cell, $F_{(1,16)} = 0.86$, $p = 0.37$; factor stimulus, $F_{(4,64)} = 21.9$, $p = 0.0001$; Interaction, $F_{(4,64)} = 1.29$, $p = 0.28$. EPSP: factor cell, $F_{(1,16)} = 0.73$, $p = 0.40$; factor stimulus, $F_{(4,64)} = 5.47$, $p = 0.0007$; Interaction, $F_{(4,64)} = 1.1$, $p = 0.41$.

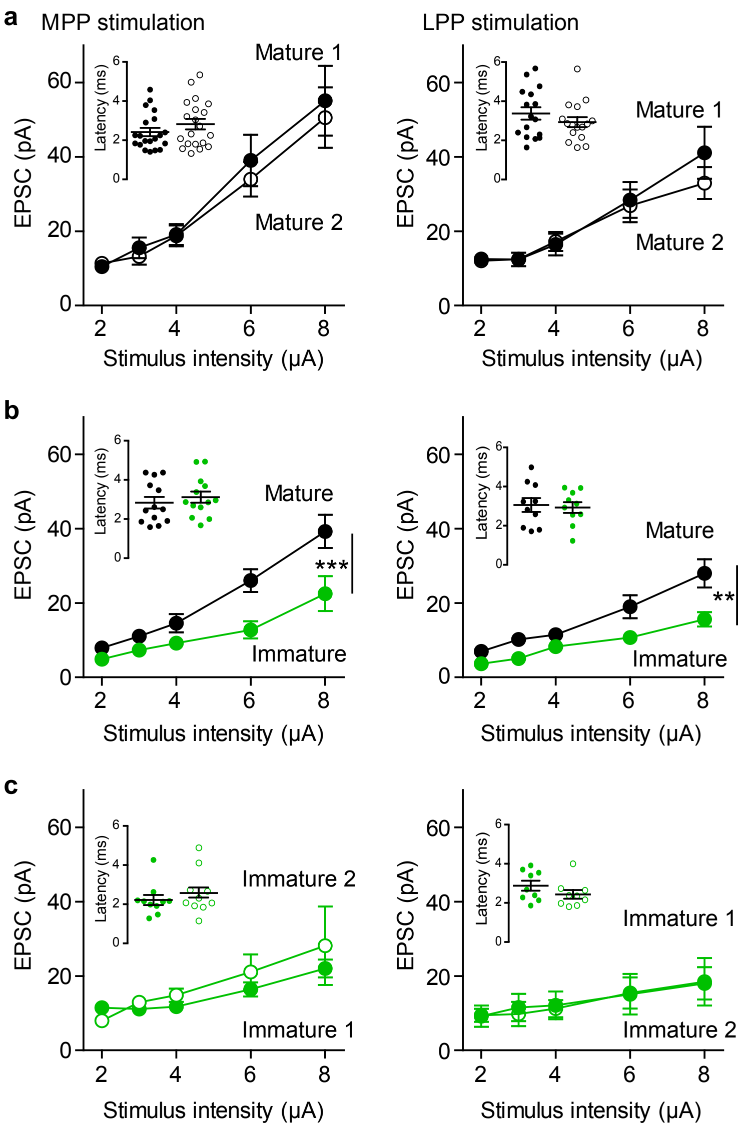

Supplementary Figure 4. Increased stimulus intensity increases the amplitude of EPSCs.

a. Increasing the stimulus intensity in the MPP (left) and LPP (right) enhanced the amplitude of EPSCs to a similar degree in pairs of mature GCs. For this analysis, failures were excluded but similar results were obtained when failures were included

(see Supplementary Figure 5). Repeated-measures two-way ANOVA, MPP: n = 20 cell pairs: factor cell age, $F_{(1,38)} = 0.57$, $p = 0.46$; factor stimulus, $F_{(4,152)} = 35.19$, $p < 0.0001$; Interaction $F_{(4,152)} = 0.16$, $p = 0.95$; LPP: n = 15 cell pairs: factor cell age, $F_{(1,28)} = 0.79$, $p = 0.38$; factor stimulus, $F_{(4,112)} = 38.1$, $p < 0.0001$; Interaction $F_{(4,112)} = 1.45$, $p = 0.22$. Insets, EPSC latencies were similar between mature and immature GCs in all conditions (paired t-tests; $p > 0.05$ for each comparison).

b. In pairs of mature and immature GCs, increasing the stimulus intensity recruited additional synaptic inputs in both cells, but the amplitude of EPSCs in immature GCs remained smaller, consistent with less innervation. Repeated-measures two-way ANOVA, MPP: n = 13 pairs: factor cell age, $F_{(1,24)} = 18$, ***$p = 0.0003$; factor stimulus, $F_{(4,96)} = 37$, $p < 0.00001$; Interaction $F_{(4,96)} = 4$, $p = 0.01$; LPP: n = 10 pairs: factor cell age, $F_{(1,18)} = 12$, **$p < 0.002$; factor stimulus, $F_{(4,72)} = 37$, $p < 0.00001$; Interaction $F_{(4,72)} = 3$, $p = 0.016$.

c. In pairs of immature GCs, increasing the stimulus intensity likewise enhanced the amplitude of EPSCs to a similar degree in both cells. Repeated-measures two-way ANOVA, MPP: n = 8 cell pairs: factor cell age, $F_{(1,14)} = 0.17$, $p = 0.68$; factor stimulus, $F_{(4,56)} = 12.17$, $p < 0.0001$; Interaction $F_{(4,56)} = 1.9$, $p = 0.13$; LPP: n = 8 pairs: factor cell age, $F_{(1,14)} = 2.05$, $p = 0.11$; factor stimulus, $F_{(4,56)} = 29.9$, $p < 0.0001$; Interaction $F_{(4,56)} = 2.13$, $p = 0.09$.

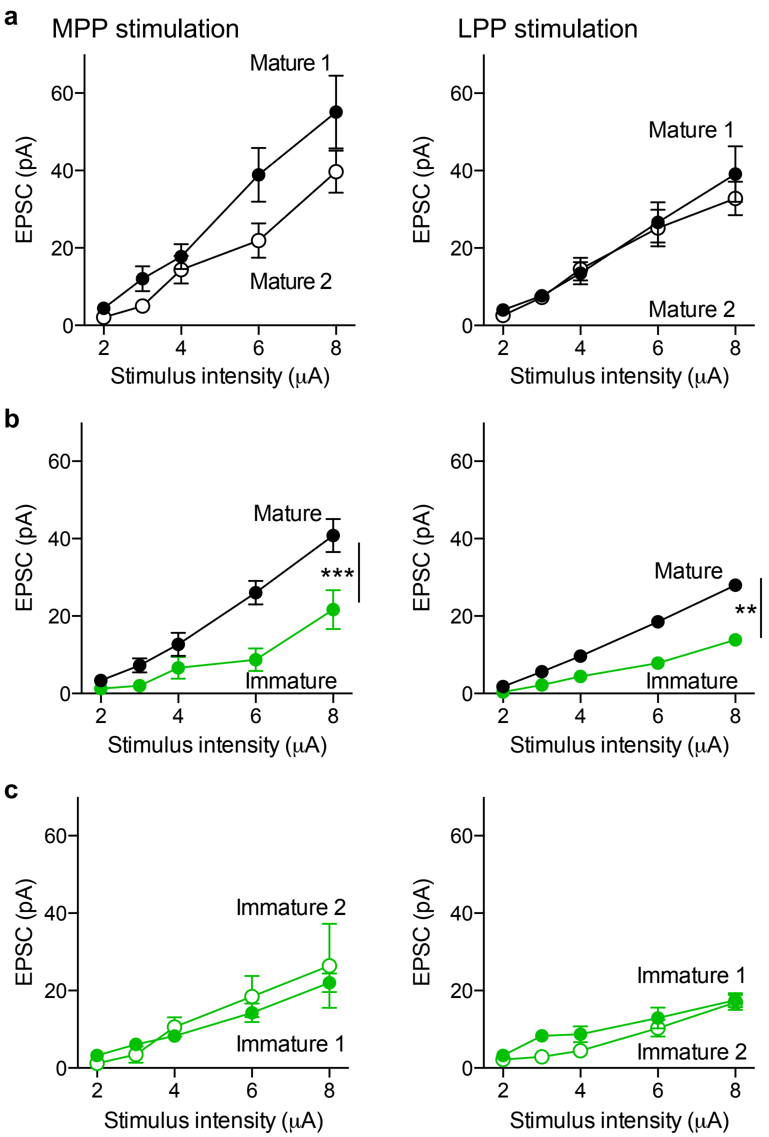

Supplementary Figure 5. Increased stimulus intensity increases the amplitude of EPSCs (failures included).

a. Increasing the stimulus intensity in the MMP (left) and LPP (right) enhanced the amplitude of EPSCs to a similar degree in pairs of mature GCs. In this analysis, all 20 trials were averaged (failures included). Repeated-measures two-way ANOVA, MMP: n = 20 cell pairs: factor cell age, $F_{(1,38)} = 3.2$, p = 0.08; factor stimulus, $F_{(4,152)} = 55.1$, p <

0.0001; Interaction $F_{(4,152)} = 1.5$, $p = 0.2$; LPP: n =15 cell pairs: factor cell age, $F_{(1,28)} = 0.24$, $p = 0.6$; factor stimulus, $F_{(4,112)} = 36.8$, $p < 0.0001$; Interaction $F_{(4,112)} = 0.35$, $p = 0.84$.

b. In pairs of mature and immature GCs, increasing the stimulus intensity recruited additional inputs in both cells, but EPSCs in immature GCs were smaller, consistent with recruitment of fewer inputs. Repeated-measures two-way ANOVA, MPP: n = 13 pairs: factor cell age, $F_{(1,24)} = 12.9$, ***p=0.0001; factor stimulus, $F_{(4,96)} = 40.3$, $p < 0.0001$; Interaction $F_{(4,96)} = 3.9$, $p = 0.005$; LPP: n = 10 pairs: factor cell age, $F_{(1,18)} = 12.4$, **p = 0.002; factor stimulus, $F_{(4,72)} = 51.4$, $p < 0.0001$; Interaction $F_{(4,72)} = 5.13$, $p = 0.001$.

c. In pairs of immature GCs, increasing the stimulus intensity likewise enhanced the amplitude of EPSCs to a similar degree. Repeated-measures two way ANOVA, MPP: n = 8 pairs: factor cell age, $F_{(1,14)} = 0.002$, $p = 0.97$; factor stimulus, $F_{(4,56)} = 15$, $p < 0.0001$; Interaction $F_{(4,56)} = 0.75$, $p = 0.56$; LPP: n=8 pairs: factor cell age, $F_{(1,14)} = 2.8$, $p = 0.1$; factor stimulus, $F_{(4,56)} = 35.8$, $p < 0.0001$; Interaction $F_{(4,56)} = 0.9$, $p = 0.32$.

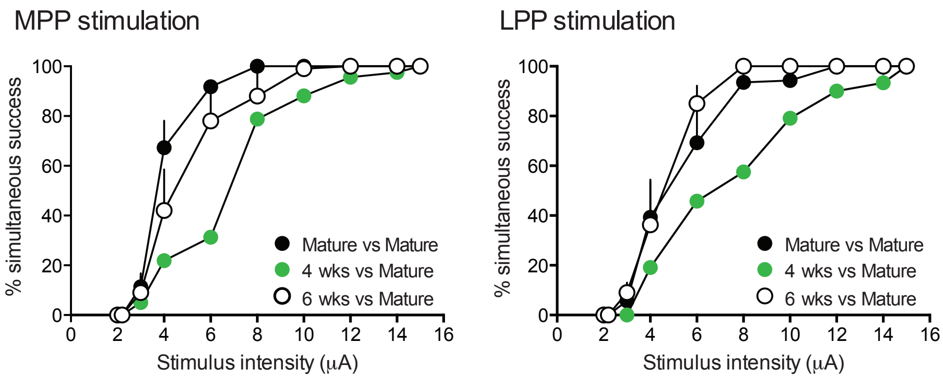

Supplementary Figure 6. Similar overlap of synaptic inputs between mature and >6 week-old immature GCs.

The % simultaneous success versus stimulus intensity is shown for MPP stimulation (left) and LPP stimulation (right) for simultaneous recordings between immature GCs at 39-52 days after tamoxifen-induced recombination (open symbols, 6 wks). Results for ~4 week-old immature (green symbols, 4 wks) are replicated from Figure 6. Repeated-measures two way ANOVA, MPP: n = 11-8-6 pairs: factor cell age, $F_{(2,22)} = 6.7$, $p = 0.005$; factor stimulus, $F_{(9,198)} = 188.3$, $p < 0.0001$; Interaction $F_{(18,198)} = 5.05$, $p < 0.0001$; Unequal N HSD post-test p(Mat vs 4 wks) = 0.007, p(Mat vs 6 wks) = 0.2, p(6 wks vs 4 wks) = 0.07. LPP: n = 7-6-6 pairs: factor cell age, $F_{(2,16)} = 5.6$, $p < 0.0001$; factor stimulus, $F_{(9,144)} = 143.1$, $p < 0.0001$; Interaction $F_{(18,144)} = 1.82$, $p = 0.02$. Unequal N HSD post-test p(Mat vs 4 wks) = 0.04, p(Mat vs 6 wks) = 0.3, post-test p(6 wks vs 4 wks) = 0.03.

Supplementary Table 1

Error	Error %-ile	Shared Mature	Independent Mature	Shared Immature	Independent Immature	p axon stim	Best NG rate	EC range @ best NG rate
0.175	0.01%	30	180	4	70	0.004	2.5%	5.25%
0.179	0.40%	29	174	4	67	0.0042	2.5%	5.00%
**0.179**	**0.56%**	**37**	**182**	**5**	**72**	**0.0039**	**2.5%**	**5.25%**
0.181	0.80%	34	180	4	71	0.004	2.5%	5.25%
0.182	1.20%	27	162	3	63	0.0044	2.5%	5.25%
0.182	1.60%	48	288	6	112	0.0025	2.5%	4.50%
0.184	2.00%	28	168	3	66	0.0041	2.5%	5.50%
0.187	3.00%	51	188	6	78	0.0035	2.5%	5.25%
0.191	4.00%	86	458	10	180	0.0016	2.5%	2.25%
0.194	5.00%	48	263	6	103	0.0029	2.5%	4.75%
0.198	6.00%	71	258	9	106	0.0027	2.5%	4.75%
0.201	7.00%	44	163	5	67	0.0039	2.5%	5.50%
0.204	8.00%	35	210	4	82	0.0037	2.5%	5.00%
0.209	9.00%	80	222	10	96	0.003	2.5%	4.50%
0.214	10.00%	52	310	6	121	0.0026	2.5%	4.50%
0.218	11.00%	94	405	12	163	0.0018	2.5%	4.00%
0.225	12.00%	52	257	6	102	0.0025	2.5%	4.75%
0.229	13.00%	64	159	8	70	0.0034	2.5%	5.25%
0.235	14.00%	80	179	10	81	0.0029	2.5%	4.75%
0.241	15.00%	60	360	7	140	0.0023	2.5%	3.75%
0.248	16.00%	70	116	9	56	0.004	2.5%	5.50%
0.257	17.00%	91	309	11	129	0.0018	2.5%	4.25%
0.266	18.00%	47	112	6	50	0.0045	5.0%	5.25%
0.274	19.00%	81	136	10	66	0.0033	5.0%	5.00%
0.286	20.00%	53	318	7	123	0.0027	2.5%	4.50%

Supplementary Table 1. Similar outcomes were generated by many fits of equation (9). The parameters used in Figure 7 are shown in bold.

Supplementary References

1 Dieni, C. V., Nietz, A. K., Panichi, R., Wadiche, J. I. & Overstreet-Wadiche, L. Distinct determinants of sparse activation during granule cell maturation. *J Neurosci* **33**, 19131-19142, doi:10.1523/JNEUROSCI.2289-13.2013 (2013).
